# Physiologic Conditions Affect Toxicity of Ingested Industrial Fluoride

**DOI:** 10.1155/2013/439490

**Published:** 2013-06-06

**Authors:** Richard Sauerheber

**Affiliations:** ^1^Department of Chemistry, University of California, San Diego, La Jolla, CA 92037, USA; ^2^STAR Tutoring Center, Palomar Community College, San Marcos, CA 92069, USA

## Abstract

The effects of calcium ion and broad pH ranges on free fluoride ion aqueous concentrations were measured directly and computed theoretically. Solubility calculations indicate that blood fluoride concentrations that occur in lethal poisonings would decrease calcium below prevailing levels. Acute lethal poisoning and also many of the chronic effects of fluoride involve alterations in the chemical activity of calcium by the fluoride ion. Natural calcium fluoride with low solubility and toxicity from ingestion is distinct from fully soluble toxic industrial fluorides. The toxicity of fluoride is determined by environmental conditions and the positive cations present. At a pH typical of gastric juice, fluoride is largely protonated as hydrofluoric acid HF. Industrial fluoride ingested from treated water enters saliva at levels too low to affect dental caries. Blood levels during lifelong consumption can harm heart, bone, brain, and even developing teeth enamel. The widespread policy known as water fluoridation is discussed in light of these findings.

## 1. Introduction 

Synthetic industrial fluoride compounds lack calcium and are listed toxic substances (Buck [1], Gleason [2], Blakiston [3], *The Merck Index* [4]). Calcium fluoride CaF_2_ is found in natural minerals and is not labeled a toxic compound because of the comparatively high lethal oral acute dose of the purified compound when tested in mammals (LD_50_ ~ 3,750 mg/kg). The fluoride compounds, sodium fluoride NaF and fluorosilicic acid H_2_SiF_6_, added into municipal water for human ingestion purposes are synthesized artificially by industrial reaction and have been used as rodenticides, insecticides, and pediculicides, with acute oral lethal doses in experimental animals comparable to arsenic and lead (LD_50_ ~ 125 mg/kg) (*The Merck Index* [4]) due to the fluoride at ~60–90 mg/kg.

Waters in the U.S. can contain natural calcium fluoride along with other calcium and magnesium salts (U.S. Centers for Disease Control (CDC) [5]), but pure pristine fresh drinking water does not contain fluoride. Fluoride is not a normal constituent of the mammalian bloodstream (*Merck manual for Health Care Professionals* [6]). It has no nutritive value [7] or physiologic function but has been believed by some to be useful for teeth based on an initial correlation with natural calcium fluoride in drinking water [1, 8]. The chief ingredient in normal teeth enamel is hydroxyapatite that contains calcium phosphate, not fluoride. After nearly 7 decades of adding industrial fluoride compounds into public water supplies in the U.S. and other countries that have agreed to this policy, the principal documented effects of ingested fluoride on teeth are to increase incidence of abnormal permanent enamel fluorosis during teeth development and to abnormally incorporate into underlying dentin bone (National Research Council (NRC) [9]). Fluorosis, unsightly at best, afflicts ~5 million U.S. teenagers aged 12–15 as of 2004 [8].

The reported adverse consequences of adding fluoride lacking calcium into public water supplies include effects on man, animals, and the environment [1, 8–12]. Ingested industrial fluoride incorporates chiefly into bone with an ion exchange process that is irreversible and thus not physiologic. Normal biochemical effects of nutrient minerals are saturable and readily reversible. *Fluorine* leads all elements in electronegativity and is extremely reactive and not found in nature. But *fluoride* is permanent because the ion has no electronegativity, cannot be reduced further, or oxidized by any known substance. Fluoride instead associates with positive charged ions in particular aluminum, calcium, and iron. Thus its toxicity depends on the environment in which it resides.

Soluble fluoride at 60 mg/kg single oral dose without calcium causes acute heart failure in research animals (CDC [5]) and caused lethal heart failure reported in a child after swallowing concentrated dental gel [13]. Twenty-five ppm artificial fluoridated water leads to chronic heart failure in research animals [5] which compares with levels during accidental overfeeds where kidney dialysis patients died (Gessner et al. [14]). At lower concentrations (~1 ppm), artificially fluoridated water supplies are documented to have caused horses, frogs, chinchillas, and alligators to die prematurely that consumed treated water continuously for extended periods of time (Spittle [12]). Discharged fluoride into the Columbia River to ~0.3 ppm blocked salmon navigation upstream to spawn (Damkaer and Dey [15]). Even though natural fluoride at 1 ppm is in the world's oceans with substantial calcium and magnesium salts, this arrangement is normal and harmless to aquatic species. 

Natural calcium fluoride is considered insoluble (to 8–10 ppm fluoride maximum depending on water pH). But industrial synthetic fluorides are fully soluble and are all toxic calcium chelators. The degree of absorption of any fluoride compound after ingestion is correlated with its solubility (Goodman and Gilman [16]). Industrial fluorides are completely absorbed, but natural fluoride minerals cryolite (Na_3_AlF_6_) or fluorite (mineral fluorspar with CaF_2_) are poorly absorbed (see Endnote 1). The dietary cations calcium and iron retard absorption by forming complexes in the GI tract. Although large populations are reported to safely consume 1 ppm fluoride in water for long periods of time, this is when it exists naturally at this level.

In what was considered unthinkable, in Hooper Bay, Alaska, in 1994 an industrial fluoridation overfeed of calcium-deficient Yukon River drinking water caused fatal heart block in an otherwise healthy 41 year old male. This is the largest known poisoning in the U.S. from a fluoridated water supply. Approximately 300 people with severe gastrointestinal pain survived the incident (Gessner et al. [14]). Electronic feeding equipment is now employed to prevent overfeeds and acute poisoning. But chronic effects of industrial fluoridation of public water supplies on humans, animals, and the environment require further study if fluoridation of fresh water supplies continues. Many countries require great expenditures to remove endogenous natural fluoride from drinking water that causes skeletal and other pathology at 8–10 ppm even when water contains substantial antidote calcium to minimize assimilation of the ingested fluoride [5]. The present study investigates conditions involved in acute and chronic fluoride toxicity and environmental effects of industrial fluorides added into public water. 

## 2. Methods

The concentration of the free fluoride ion was mathematically computed at which the solubility of calcium fluoride would be exceeded with calcium concentrations known to be physiologic. [F^−^] = (K_sp_/[Ca^2+^])^1/2^ from the definition of the solubility product constant for insoluble salts where CaF_2_ → Ca^2+^ + 2F^−^ and K_sp_ = [Ca^2+^][F^−^]^2^. Consideration was made for physiologic temperature by using the published K_sp_ at 37°C.

 A 0.9 ppm fluoride solution in distilled water was measured for the free fluoride ion concentration over a wide concentration range of added calcium ion from aliquots of a calcium biphosphate solution. In other experiments, a 1.2 ppm fluoride concentration solution was measured for free fluoride ion level as a function of pH. Acidity was adjusted with dilute acetic acid. All readings were made at room temperature with a LaMotte Instruments fluoride ion specific electrode calibrated with a 1.00 ppm fluoride standard solution in distilled deionized water. The electrode was rinsed with the solution to be tested for each measurement. The instrument reported accurate readings for known standard solutions within ±0.05 ppm fluoride over the temperature range 15–30°C.

## 3. Results/Discussion

### 3.1. Acute Toxicity

Acute fluoride poisoning in man is not rare (Goodman and Gilman [16] p. 804). The concentration of fluoride is here calculated that would cause calcium fluoride precipitates to first form from the known solubility product constant for calcium fluoride (K_sp_ = 8 × 10^−11^ at 37°C) and the known concentration of calcium ion in normal human blood (2.2 mM). The K_sp_ varies slightly with temperature and may be computed at 37°C (310 Kelvin) from the relation ln⁡(K_sp_) = −ΔG/(RT) (Lide [17]) for calcium fluoride with the free energy for the dissociation of calcium fluoride ΔG = 59 kJ/moL and K_sp_ = 3.4 × 10^−11^ at 25°C (298 K).

The computed fluoride level at which an aqueous solution containing physiologic calcium (3 mM) at physiologic temperature (37°C) is precipitated is 0.11 mM fluoride (2 ppm). The concentration of blood fluoride where the blood calcium level would be lowered to the lethal low level of about 1 mM is 0.2 mM fluoride (3.8 ppm). This also compares with measured blood fluoride levels in man (2-3 ppm) due to acute lethal poisonings after accidental ingestion of soluble fluorides (Teitz [18] p. 1130). 

The calculated calcium levels that would coexist in fluid with a given fluoride level from solubility considerations were compared with actual measurements of blood levels of calcium and fluoride ion in victims of fluoride poisoning (Gessner et al. [14]) in Hooper Bay. The calculated theoretic fluoride levels that would lower prevailing blood calcium levels compare closely with the actual fluoride levels measured in the blood of victims poisoned with fluoridated municipal water. The victim who died of heart failure from fluoride had a measured fluoride level of 0.18 mM, while another victim that survived had blood fluoride at 0.48 mM. These concentrations of fluoride from solubility considerations produce calcium ion lowering to levels reported to decrease beat rates in isolated mammalian heart cells (Wang et al. [19]).

The fact that fluoride lethality occurs at concentrations known to compare with saturation and activity reduction for calcium ion brings forth an aspect of fluoride toxicity that is counterintuitive. Ionized calcium levels in human plasma can vary in some cases from 1.5 mM in hypocalcemia to 4.5 mM in hypercalcemia (as in hyperparathyroidism or excessive Vitamin D intake) (Davidsohn and Wells [20]). The assimilation of ingested fluoride is drastically minimized by calcium ion in the gastrointestinal tract [5] and calcium is thus the recognized antidote to fluoride poisoning. This would suggest that individuals with higher blood calcium would be more resistant to fluoride toxicity. But K_sp_ calculations indicate that higher blood calcium levels require less blood fluoride to reduce calcium activity or mobility. 

**Figure 1:**
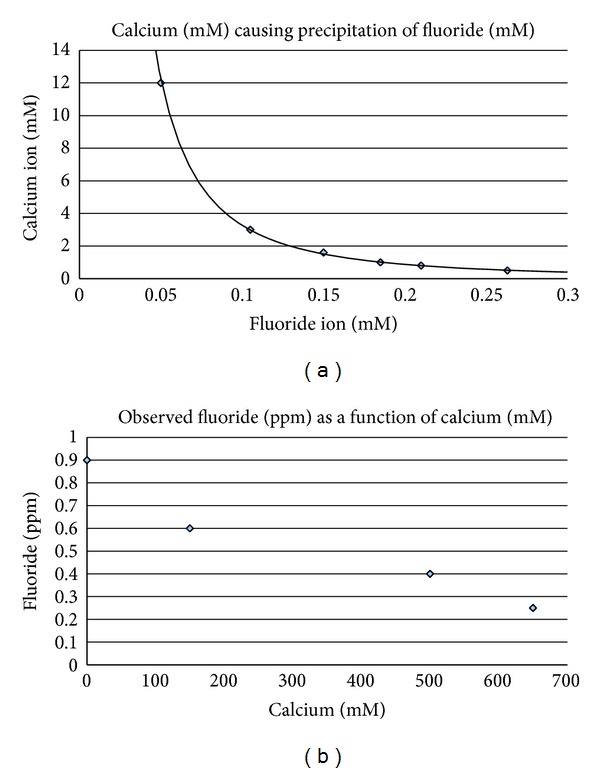
(a) *Calcium concentrations (mM) that precipitate various fluoride concentrations*. From the known solubility product constant K_sp_ for calcium fluoride, the calculated isocurve for CaF_2_ precipitation is shown as a function of varying concentrations for the two ions. Calcium levels in normal blood (1–3 mM) reach precipitation maximum solubility with 0.1–0.18 mM (1.9–3.4 ppm) fluoride. For 3 mM calcium, precipitation begins to occur when fluoride is 3% of calcium and for 1 mM at 18%. The Hooper Bay poisoning incident produced a lethal 5 ppm blood fluoride level in one victim. Whether it is a low Ca/F ratio or rather calcium fluoride saturation that is required for acute toxicity is not known. (b) *Calcium effects on measured fluoride ion concentration*. A 0.9 ppm fluoride solution in distilled water was measured for free fluoride ion level with a LaMotte fluoride specific electrode calibrated with 1.00 ppm sodium fluoride in distilled deionized water at room temperature. Calcium ion was adjusted over a wide range by addition of aliquots of calcium biphosphate. Fluoride readings progressively decrease with increasing calcium concentration as expected over the range 30–650 mM.

Figure 1(a) indicates the calcium fluoride precipitation isocurve over a broad concentration range for the two ions. The ions are precipitated when present together at any ordered pair of concentrations indicated along the curve. Lower blood calcium levels require higher fluoride blood levels to begin precipitation. The effect is quite substantial in varying from 2.5 to 5 ppm fluoride lethal levels for subjects with 4.5 and 1.5 mM calcium, respectively. This may help explain the broad variability in reported blood fluoride levels causing toxicity and why calcium can exist within a normal range during acute fluoride poisoning from ingestion in humans.

Wang et al. [19] also found that the heart cell beat rate in cultured cells in well-controlled experiments progressively slows with increasing fluoride levels in a concentration-dependent manner. Beat rates were slowed 17% by 0.15 mM (2.8 ppm) fluoride, a level that precipitates physiologic concentrations of calcium in solution. Unlike skeletal muscle, cardiac muscle requires extracellular calcium ion from the bloodstream to couple electrical excitation of the cell membrane with contraction of cardiac muscle fibers. Each time the heart contracts, calcium fluxes into the heart cells from the extracellular fluid. When the heart relaxes, the calcium is pumped back out of the cell allowing the fibrils to relax. Lowered extracellular calcium ion levels block contraction of the heart.

Blood with high ionic strength is quite different than aqueous solutions in a laboratory. Calcium fluoride precipitates have not been found generally throughout interstitial fluid in cases of acute lethal fluoride poisonings. These data taken together suggest that fluoride ingestion is lethal by causing decreased activity of the free calcium ion and blockage of heart contractions. Fluoride acute toxicity has long been known to be accompanied by increased blood potassium levels (Burgstahler [21]), where membrane sodium potassium ATPase is also inhibited by fluoride or low calcium. Therefore, the sequential mechanism of lethality cannot be precisely stated.

Fluoride tends to associate with and bind calcium ion. And calcium is concentrated throughout the body including teeth, bones, ligaments, aorta, skeletal muscle, and brain [5, 10, 16]. But the most crucial physiologic function requiring calcium that is sensitive to industrial fluoride at acute levels is the beating heart. It is well known that extracellular calcium is an obligatory requirement for heart cells to undergo contraction after electrical excitation. Heart cells do not have a well-developed sarcoplasmic reticulum to store calcium for this purpose as does skeletal muscle which does not exhibit this extreme sensitivity to changes in blood calcium. The cellular uptake of calcium occurs during the plateau phase of the cardiac action potential. Extracellular calcium is necessary for the development of contractile force. The strength of contraction (inotropic state) of the heart depends on extracellular calcium with half-maximal contractility at 0.5 mM.

### 3.2. Chronic Toxicity

Kidney dialysis patients have frequently been killed from fluoridated water during accidental fluoride overfeeds [14] because dialysis units are not equipped to eliminate fluoride from blood [22]. Even more alarming are kidney patient lethal fluoride poisonings from a form of congestive heart failure if hemodialysis units use fluoridated water containing targeted concentrations of fluoride. As pointed out by Dr. Ahmad, Medical Director, University of Washington, Seattle, hemodialysis patients receive more than 400 liters of water weekly and fluoride levels above 0.2 ppm if not pre-cleaned cause significant morbidity and mortality [22]. Fluoridated water at 0.7–1 ppm is unsuitable for dialysis and the FDA has published instructions to that effect. Deaths have even occurred because fluoride-removing deionizer resins when full leached fluoride back into the water used for dialysis.

The mechanism by which fluoride from blood at desired lower levels irreversibly accumulates in bone (NRC [9]) does not involve precipitation of ionized calcium. Fluoride is then below the K_sp_ for direct precipitation. Instead, an ion exchange mechanism occurs at extremely minute fluoride levels where the fluoride ion merely by diffusion exchanges with hydroxide on bone hydroxyapatite [16]. The precise formula unit of bone matrix has been variably described as calcium phosphate hydroxide Ca_3_(PO_4_)_2_Ca(OH)_2_ or pentacalcium monohydroxy orthophosphate Ca_5_(OH)(PO_4_)_3_, or Ca_10_(PO_4_)_6_(OH)_2_ (*The Merck Index* [4]). Very different lattice packing and fluoride sensitivities occur among spongy bone, compact bone, teeth dentin, and hard enamel. 

A fluoride ion solution made in soft or distilled water has a higher chemical activity or chemical potential compared to the activity of the ion at the same concentration when accompanied with calcium or magnesium in solution. Although much less sensitive and exquisite than an actual biological cell membrane, a fluoride specific electrode senses such a difference. Fluoride electrode measurements of a solution of sodium fluoride fixed at 0.8 mg/L (ppm) (0.042 mM) in deionized water were examined at various calcium levels over a wide range. Thirty mM calcium and higher cause substantial inter-ionic interactions with fluoride that significantly lower diffusion or Brownian motion of the fluoride ion because of the relatively massive divalent positive charge on the compact calcium ion. Further addition of calcium to 650 mM causes progressive decreases in the free fluoride ion measured level due to precipitation of calcium fluoride particles that the electrode cannot detect. The calcium level theoretically calculated to first begin fluoride precipitation for 0.9 ppm fluoride is approximately 30 mM which is consistent with Figure 1(b) observed data.

The phenomenon of fluoride-induced decreased chemical activity (i.e., in the absence of precipitation) applies to Group II cations including magnesium ion prevalent in all foods and natural hard waters. In contrast, fluoride accompanied in solution with Group I metal cations, such as sodium or potassium, exhibits no decline in activity over a broad range of cation concentration (data not shown), because these ions are only monovalent in charge. 

Activity coefficients for the fluoride ion are substantially reduced in the presence of calcium and magnesium divalent cations (Moore [23]). This effect may be compared to the phenomenon of attraction between fluoride ion and hydrogen atoms in water known as hydrogen bonding which decreases the Brownian motion and diffusion of the ion. These factors determine the overall biologic effect of fluoride ion in living organisms. Calcium decreases assimilation through the gastrointestinal tract but in the bloodstream lowers the fluoride required for calcium sequestration. Further, membranes exhibit complex structural and functional features that are modulated by calcium and magnesium, including region-specific effects on membrane lipid fluidity (Sauerheber and Gordon [24]). Fluoride diffusion from a solution rich in calcium ion may be impaired even though far below the level required for binding as calcium fluoride precipitate. This electrical attractive force is also responsible for the fact that fluoride at levels below the K_sp_ is trapped in bone by ion exchange. 

**Figure 2:**
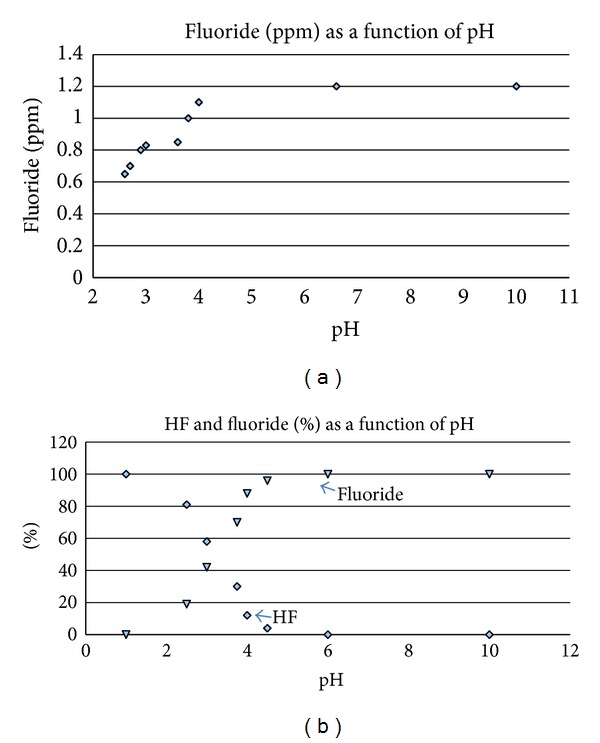
Fluoride protonation depends on prevailing acidity following the equilibrium reaction F^−^ + H^+^↔HF. (a) All measurements of fluoride ion concentration were made with a LaMotte Instruments fluoride ion specific electrode calibrated with a 1.00 ppm fluoride standard solution in distilled deionized water at room temperature. Readings for the 1.2 ppm true concentration solution progressively decrease as pH decreases. Acidity was adjusted with dilute acetic acid. Stomach acid pH normally varies from 1.5 to 3 (Teitz [18] p. 1072), where fluoride is mostly protonated as hydrofluoric acid HF. (b) The percent contribution to the total fluoride from the free ion F^−^ (triangles) and intact HF (diamonds), calculated theoretically from the Henderson Hasselbach equation pH = pK_a_ + log⁡{[F^−^]/[HF]} over the pH range 1–10, utilizing the known K_a_ for HF. HF decreases while F^−^ increases from pH 1 to 5. At pH 3.14 (= pK_a_ = −log 7.2 × 10^−4^), HF is half dissociated.

As shown in Figure 2, changes in pH affect the percent of fluoride that converts to HF. As HF, fluoride gains entry into the bloodstream because HF is a neutral small molecule comparable in size to the water molecule and is freely permeable through the biologic membrane (Whitford et al. [25]). The K_a_ for HF indicates that it is a weak acid. As a small molecule, HF is the most penetrating corrosive. Its assimilation is most efficient at stomach pH in the absence of substantial calcium. 

Industrial fluoride in drinking water can cause GI distress in human subgroups because fluoride converts to HF in the stomach (NRC [9], p. 268). Even at low concentrations HF can aggravate and prevent healing of ulcerated tissue. Industrial fluoride in drinking water at gastric pH is mostly protonated HF. At pH 2, 96% of fluoride is HF (NRC [9]) in agreement with calculated levels in Figure 2(b). Structural damage to mucosa is detected at 20 ppm HF in 15 minutes. Lower concentrations cause pain without visible damage. Human case studies proved that abdominal discomfort occurs from drinking 1 ppm artificially fluoridated water (Waldbott [10]; Petraborg [26]). In a careful placebo controlled double blind clinical study, 1 ppm fluoridated water caused GI discomfort in 7% of subjects (Grimbergen [27]). Exposing the GI tract to HF from the duodenum to the Ampulla of Vater before reneutralization is a false practice. The more elderly the person with slower gastric mucosa turnover, the more likely symptoms can develop. In the presence of ulceration or gastric carcinoma, industrial fluoridated water must be avoided. Acute pain requiring hospitalization in Hooper Bay reflected gastric HF at ~50–100 ppm.

HF at high concentrations dissolves glass and permeates porcelain. This is not due to the hydrogen or the fluoride ions since strong acids do not have this ability and soluble fluoride salts slowly etch glass. Corrosiveness of HF is due to its extremely tiny uncharged covalent structure, intermediate between fluorine F (0.8 Å^3^, 128 pm diameter) in F_2_ and the 10-fold larger volume fluoride ion F^−^ (7.9 Å^3^, 272 pm diameter) in ionic compounds. As a 2nd period nonmetal, fluoride at any concentration forms abnormal interatomic hydrogen bonding (H^+^- -F^−^) and disrupts normal intermolecular hydrogen bonding (H^+^- -N or H^+^- -O) in water and macromolecules. It is a general enzyme inhibitor in some cases at 0.2 ppm (Yiamouyiannis [11]), the targeted blood level reported in residents of 1 ppm fluoride water areas (NRC [9] p. 70).

### 3.3. Natural and Industrial Fluoride in Water

Dental officials report in CDC fluoridation literature that fluoride ion is identical in natural and industrial compounds. This is correct. However, the assimilation of ingested fluoride is minimized by calcium in the GI tract (CDC [5], Goodman and Gilman [16]). The conversion of fluoride to HF measured with an electrode is also reduced in the presence of calcium at levels far below saturation (data not shown). Natural fluoride is accompanied with antidote calcium to prevent acute lethality and reduce chronic toxicity. Nevertheless, the CDC goes on to argue that the relative safety of water containing calcium fluoride at 1 ppm is sufficient proof that industrial fluoride at 1 ppm will exert no significant pathology. But toxic effects of natural fluoride in water can often be difficult to measure, such as widely reported effects on human brain function [8]. And any effect would not be identical for water treated with an equal level of industrial fluoride which is assimilated more efficiently [5]. Indeed, salmon are unaffected by natural 1 ppm fluoride in ocean water where calcium is extremely high but are narcotized by industrial fluoride in soft water at only 0.3 ppm (Damkaer and Dey [15]). Thus, the mere absence of gross observable bone abnormality from natural fluoride at levels below the Environmental Protection Agency Maximum Contaminant Level (EPA MCL) of 4 ppm should never have led to the presumption that lifelong consumption of infused industrial fluoride at any purported concentration (>0) would have no possible pathologic consequence.

 From Figure 1, it is evident that at 30 : 1 calcium to fluoride, the assimilation from the GI tract, if compared to conditions in a laboratory, would begin to be impaired. Waters in the U.S. average 50 ppm calcium to 0.2 ppm fluoride naturally (100 : 1). But soft waters allow more efficient absorption. The ratio of calcium ion molarity (around 0.12 mM or 7 ppm) to infused total fluoride molarity (0.05 mM or 1 ppm) in soft water regions in an artificially fluoridated city is very low. Hard water regions are more protected from fluoride assimilation. Fluoride toxicity depends on its environment.

Sources of ingested industrial fluoride are diverse. The fluoride that is absorbed into the bloodstream arises chiefly from public water supplies. But substantial amounts are assimilated also from foods, toothpaste (NRC [9]), mouth rinses, and some bottled waters. The EPA currently allowed levels in drinking water in the U.S. were found by the NRC to not be protective of human health [9]. The plethora of chronic effects is accompanied with alterations in calcium homeostasis where bone fluoride is retained for an estimated 20 years (NRC [9] p. 133) which affects calcium mobilization from bone into blood. Variations in biologic outcome of course occur because of differences in water hardness, diet, and whether it is natural insoluble fluoride or artificially infused soluble fluoride without calcium.

Assimilation of industrial fluoride from water into the bloodstream in humans can be seemingly well-tolerated for long time periods because bone efficiently traps the calcium chelator from interstitial fluid to minimize exposure to soft tissues. Bone is the final resting site for 95% of all retained fluoride. Even there the intrusion causes increased osteoblastic activity [9, 16] in response. Fluoride accumulates from consumption in a 1 ppm fluoride water region, in the absence of other known sources, to 2,500 mg/kg in two years and to 3-4,000 mg/kg lifetime [9]. Bone weakening occurs around 3,500 mg/kg. Bone pathology from ingested water fluoride has been widely described (NRC [9]; Connett et al. [8] appendix 2) consistently in research animals. In spite of variations in water hardness and the fact that man cannot be controlled for variables as can research animals, more human studies correlate 1 ppm fluoride ingestion with bone weakening than studies that do not. Before bone weakening occurs, the abnormal incorporation of fluoride that is irreversible affects calcium whole body metabolism. In the normal cell, a steep calcium concentration gradient varies from millimolar levels outside in interstitial fluid to micromolar levels in cell cytoplasm. Magnesium exists at millimolar concentrations on both sides of the membrane. Fluoride ion has no functional purpose in the cell but being attracted to these ions would affect their chemical activity. 

The chemical characteristics of fluoroapatite in living bone tissue are not clearly understood. But fluoride accumulation, being non-saturable and irreversible, is pathologic not physiologic. Skeletal fluorosis poisoning from long-term uptake involves fluoride sources with concentrations that are obviously below that which would cause acute lethal poisoning. In contrast, there are no fluoride blood levels low enough to prevent incorporation into bone. Since fluoride is not a normal body component, there are no endocrine mechanisms to mobilize fluoride from bone after binding. Although calcium fluoride is a “weak acid salt” because it readily dissolves in acidic media, extracellular fluid is alkaline pH 7.4. It is thus not surprising that fluoride cannot be removed from bone. As an insidious poison it can affect calcium homeostasis as it accumulates during lifelong exposure (see Endnote 2). The accumulation can slowly progress from bone weakening (at ~3,500 mg/kg) to arthritis-type bone pain (~7,000 mg/kg) and, in some regions of the world with high endemic fluoride in water, to total immobility (~10,000 mg/kg) [8, 9]. At any stage, there is no cure because fluoroapatite at extracellular pH is insoluble. Before symptoms appear, fluorosis can only be detected by expert X-ray analysis and is associated with bone being more subject to fracture.

Teeth enamel is very much different than hydroxyapatite in dentin and skeletal bone. Fluoride cannot incorporate into translucent crystalline enamel as it efficiently can in opaque underlying bony dentin. Once fluoride enters the bloodstream and then bone tissue, its chemistry is pathologic since the fluoride has entered the sanctity of a living organism. Stages II and III skeletal fluorosis have historically been considered absent in the U.S. population (NRC [9]). There is no reason to believe that this will remain as long as more cities require fluoridation and more sources of fluoride exposure continue to expand. Fluoridated drugs containing C–F bonds that are partially metabolized and intended for lifelong ingestion, such as some statins, are of concern.

In elder years with fluoride-loaded bone [8], continuous consumption of fluoride with reduced binding sites available in bone leads to accumulation in soft tissues, including brain [8] and in ligaments, tendons,and aorta [16]. Fluoride can cross the blood-brain barrier in man [11] and is found in virtually all tissues [16] but concentrates in bone, thyroid, aorta, kidney [16] and pineal gland in the brain [8]. This is perhaps by passage as trace neutral HF comparable in size to a water molecule. In animal brain (Reddy et al. [28]), this blood level causes direct histopathology observed by electron microscopy. 

Long-term exposure appears to decrease IQ in children (Connett et al. [8] pp. 148–156) even from natural fluoride in water. Alum used as a clarifying agent in public water systems produces residual aluminum ion (~0.05 mM). Fluoride complexes with aluminum in the acidic stomach and is assimilated. Binding of aluminum to abnormal brain proteins in Alzheimer's and in mammals that causes pathologic effects (Varner in: [8, 9]) indicates caution in consuming aluminum fluoride from water in the presence of brain abnormality. There are no cures for either bone fluorosis or brain degradation. Thus, yearly incidence of new cases adds directly to net prevalence for these conditions. The U.S. has 1/3 million hip fractures in the elderly annually [8] and lethal Alzheimer's cases have been steadily rising for decades (see Endnote 3). 

Systemic fluoride at sub-acute levels incorporates into atherosclerotic plaque in coronary vessels of cardiovascular disease patients directly revealed with PET scans in a study at the Veterans Administration Healthcare Center, Los Angeles (Yuxin et al. [29]). Fluoride is accumulated by the aorta and concentrations increase with age that reflect calcification that occurs in this artery [16]. The extent is determined by water hardness and all sources of fluoride exposure. Chronic ingestion of subacute concentrations of fluoride from drinking water weakens heart muscle in animal studies (CDC [5]) and can cause alterations in heart function in humans (Varol et al. [30, 31]). Per capita cardiovascular deaths increased after Grand Rapids, MI, and Newburgh, NY, began industrial fluoridation (U.S. Public Health Service Congressional Record, Mar 24, 1952). 1,059 heart disease deaths occurred per year in 1948 in Grand Rapids, MI after 3 years of fluoridation but 585 yearly before fluoridation. The NY News, Jan 27, 1954 reported after 9 years of fluoridation in Newburgh, NY there were 882 heart deaths per 100,000 which was 74% above the National average. Increased incidence of EKG abnormalities was reported to occur in patients having tooth fluorosis in high natural fluoride areas (Xu et al. [32], Takamori et al. [33]). 

Ironically, the level of fluoride in saliva that filters from the bloodstream after swallowing water with 1 ppm fluoride is a miniscule 0.02 ppm average ([9] p. 71, personal communication K. Theissen, co-author of NRC Report). This is unable to influence teeth caries at 75,000 times lower concentration than in toothpaste at 1,500 ppm. Consistent with this, the largest epidemiologic study in the U.S. found caries incidence does not correspond to fluoride levels in water (Hileman [34]). The largest international study indicates that caries incidence is lowest in cities with the lowest levels of water fluoride and with calcium-sufficient diets (S. P. S. Teotia and M. Teotia [35]). The U.S. CDC [36] published that systemic blood-borne fluoride from swallowing does not affect dental caries. In fact, systemic fluoride plays the most major role in causing the current U.S. high incidence of tooth fluorosis in children that prompted the U.S Health and Human Services to request in 2011 that water added fluoride be lowered from ~1 ppm to 0.7 ppm. But this is not expected to eliminate the problem. 

Normal teeth enamel is a calcium phosphate matrix that does not contain fluoride. Research animals raised under controlled conditions in the complete absence of any fluoride source, either natural or synthetic, develop normal teeth enamel without increase in incidence of spontaneous dental caries (reviewed in: Yiamouyiannis [11]). These controlled experimental data carefully using the scientific method demonstrated in three separate laboratories that ingested fluoride is not a mineral nutrient. In another animal study 1 ppm artificial fluoridated drinking water did not decrease incidence of spontaneous dental caries. Thus fluoride does not affect teeth caries by either a systemic mechanism after assimilation or by direct contact with teeth surfaces from either fluoridated saliva or from treated water in the tested animals.

 This enables one to understand the original correlation proposed in the U.S. between *natural* calcium fluoride in water and human caries incidence that attributed causation to fluoride. The biologic essential minerals calcium, which builds strong teeth and bone and magnesium totaled 302 ppm in the hard water at the time. Furthermore Ziegelbecker found the original correlation was mere scatter and that no relationship existed between caries incidence and water fluoride concentration when all data are considered (Connett et al. [8] p. 50). Fluoride in drinking water whether natural or unnatural has no functional purpose. In fact this statement was published in the textbook written by dentist Dr. George Heard who first proposed to the Public Health Service in 1950 the idea that natural fluoride in water might be of benefit for tooth decay. He apologized later for the extrapolation in a letter to the U.S. Health and Human Services [37] after finding that children raised on water with fluoride developed crumbly teeth interiors. This is from fluoride incorporation into underlying bony dentin.

Water districts most commonly now inject *unnatural* fluoride compounds into water to increase blood fluoride levels in consumers (personal communication, CA Department of Public Health official). The toxic hazardous waste produced from fertilizer manufacturing, fluorosilicic acid H_2_SiF_6_, is the most widely used substance (Connett et al. [8]; EPA [38]). This was after an EPA official in 1983 described the direct use of this waste as a source of fluoride for drinking water supplies. Controlled human clinical trials for safety and effectiveness have never been completed with water treated with either sodium fluoride or fluorosilicic acid. The U.S. Food and Drug Administration has never approved fluoride compounds for ingestion in the U.S. The FDA has written that fluoride is not a mineral nutrient and has labeled fluoride in water an uncontrolled use of an unapproved drug (Lovering [7]). In 1966, the FDA banned the sale of fluorides intended for ingestion by pregnant women due to lack of effectiveness on dental health in offspring (Horowitz and Heifitz [39]). 

Early interpretations of data from Newburgh, NY and Grand Rapids, MI where synthetic industrial sodium fluoride was first infused into public water supplies in 1945 were flawed, as pointed out by the dentist and academic statistician Sutton [40]. Assimilated fluoride in the treated cities caused delayed teeth eruption where missing teeth were improperly scored as absence of cavities. Unbiased double blind methods were not used. Caries incidence in control cities were not assessed synchronously, among other unanswered problems. Bone cortical defects were attributed to fluoride assimilation from the treated water supply [8]. 

Recent studies indicate that rather than strengthening teeth, fluoride water consumption during teeth development decreases calcium content in teeth with electron microscopic structural abnormality and weakening (Susheela [41]). Ingested fluoride abnormally incorporates substantially into underlying dentin (NRC [9]). Ingested fluoride is of course no value to adult teeth [16].

### 3.4. Other Fluorosilicic Acid Products in Water

Fluorosilicic acid is not a source for fluoride in any natural water supply. Its infusion quantitatively adds three ingredients: fluoride, sodium, and silicic acid. Fluoride and sodium are not components of pristine fresh drinking waters such as in the Pacific Northwest. Neither fluoride nor silicic acid are listed in the Merck Manual for Health Care *Professionals* [6] or in Clinical Chemistry (Teitz [18]) texts as constituents of normal human blood because neither are mineral requirements. 

Silicic acid H_4_SiO_4_ from fluorosilicic is typically 0.6 ppm when fluoride is adjusted to 0.8 ppm. Silicic acid with its low dissociation constant remains the intact molecule at alkaline pH and may be the ingredient that leaches lead salts from lead-based plumbing that does not occur with sodium fluoride. The EPA has no MCL for silicic acid because it is historically believed to be harmless where found in water supplies. Artificial silicofluoridation of municipal water supplies however caused alligators to develop liver silicosis that did not occur in alligators given water without fluorosilicic acid [12]. NMR studies confirm that fluorosilicic acid fully dissociates into fluoride ion and silicic acid at community water pH but forms a silicofluoride complex at pH 3 (identified as SiF_5_^−^) (Finney et al. [42]) as in stomach acid. A positive view has been presented for possible benefits of silicic acid consumption to cause soft fingernails and changes in skin structure (Barel et al. [43]) due to stimulation of collagen formation by fibroblasts. But this effect may be nonphysiologic. Long-term effects on teeth enamel have not been studied even though silicic acid is used in agriculture to degrade calcium phosphate in soils.

**Figure 3:**
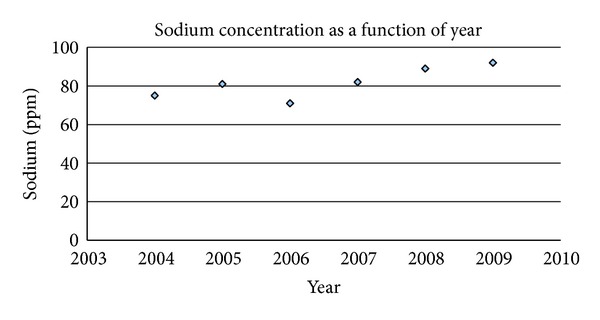
Data are from public published water quality reports from the Metropolitan Water District, Los Angeles, for sodium as a function of year. The concentration increased progressively after 2007 when industrial fluorosilicic acid with caustic soda injections began. Every 24 tons of fluorosilicic acid require 14 tons of sodium hydroxide to maintain pH at 8.4 (two H^+^ ions from H_2_SiF_6_ require two sodium ions). Sodium at 116 ppm has been found to decrease yields and affect vegetable and fruit quality. Sodium is released into the Colorado River by scores of industries lining the river. The EPA Salt Abatement Program limits releases from companies to one ton daily each, but the existence of numerous sites have led to this level. The EPA secondary standard for TDS (500 ppm) is exceeded but is not enforced—plants can tolerate natural TDS from 800 to 1000 ppm. No MCL has been developed by EPA for sodium, since fresh water has historically been low in sodium. Sodium is absent from pristine fresh drinking water, and the National average is 15 ppm. The Colorado River winds 700 miles from Western Wyoming to Southern California and the Mexico border. The speed of the river flowing at a typical width of 900 ft. is 4.5 ft/sec. with an estimated travel time of 9.5 days along its course. The source of fluoride along its length that produces the fluoride level of 0.2 ppm is presumably natural but has not been identified.

In Figure 3 note that Southern California public drinking water sodium levels increased from an average 80 ppm prior to artificial fluoridation to an average 93 ppm after 2007 when fluorosilicic acid injections began that required sodium hydroxide to neutralize acidity. Many plant species that have thrived in this region including the widely cultivated avocado are saline intolerant (Musyimi et al. [44]). Elevated sodium in irrigation water can alter avocado leaf number, chlorophyll content, chloride content, root weight, and transpiration water loss rate. At 345 ppm sodium, chlorophyll content is reduced in leaves by 40%, chloride content by 42%, and transpiration rate of water loss 21% after only 7 days with sodium at this level. In January, 2011 when the Health and Human Services recommended that infused fluoride not exceed 0.7 ppm in public water supplies [45], Metropolitan Water followed this request in Southern California and the sodium level dropped back near 85 ppm.

### 3.5. Interpreting Fluoridation Policy

Federal water laws were enacted by the U.S. Congress for the protection of the Nation's waterways that often cross State boundaries. The Water Pollution Control Act conceived by President John F. Kennedy states that its mission is to maintain the natural chemistry of the Nation's water. The Safe Drinking Water Act provides an exception that allows additives that sanitize drinking water but otherwise prohibits any National requirement for the intentional incorporation into water of other substances regardless of perceived benefit. This includes foods, supplements, nutrients, drugs, poisonous substances, or pollutants. Together with the WPCA, this also includes ingredients that may be present naturally in one particular water that are absent or at lower levels in other waters. Natural U.S. waterways are a heritage that are under Federal protection. The SDWA also stipulates that States can be no less restrictive. 

In practice, it has proven difficult to follow Federal water law in its entirety because of human industrial and other activities. In the case of “fluoridation,” Oral Health Division officials within the CDC endorse and encourage ingestion of fluoride from water at dictated concentrations. The beliefs are that it is a systemic dental treatment and that natural calcium fluoride contaminant in some waters might suggest it is a desired water ingredient. Unable to mandate a Federal requirement, the CDC encourages States to do so. Some States legislate “mandatory fluoridation.” But this does not consider the requirements of all Federal water law taken together and can thus be argued to be invalid (Balog [46]).

 The Congress assigns to the FDA full authority to regulate use of any oral ingested substance to treat humans including nutrients, approved drugs, unapproved drugs, and substances used as drugs or treatments, regardless of method of dissemination. As mentioned, the FDA banned the sale of fluoride compounds intended to be taken internally by pregnant women. Yet industrial fluoride is consumed by the general public in those 70% of all districts that artificially fluoridate water. 

No Federal agency accepts liability for the unnatural fluoride infusions (Connett et al. [8]). The FDA wrote that added fluoride is a contaminant for regulation by the Environmental Protection Agency [46] and does not require labeling fluoride content of bottled water to avoid public perception that fluoride might be thought to be a normal ingredient in water. The EPA considers intentionally added fluoride a water additive and accepts no authority for its regulation and allows states and cities to fluoridate. Yet, EPA offers assistance in the use of chemicals certified by the private organization the National Sanitation Foundation [8]. The NSF denies liability for use of fluorides [47] and does not publicly disclose all safety or effectiveness data for its use as a water additive. The CDC endorses the practice but shuns liability and regards fluoride in water as a supplement ingredient for teeth. Only the FDA is authorized to regulate dietary supplements. 

Water districts rely on State Health Departments for safe conditions of use. These departments are under the CDC and also refuse liability and assign liability to the cities themselves. Litigation over the Hooper Bay incident pitted State versus city disputing liability. The EPA and CDC advise compliance with NSF Standard 60 requirements for fluoridation chemicals. But NSF neither discloses its detailed composition (for proprietary reasons) nor performs toxicity testing (Connett et al. [8] p. 26–28). NSF labels fluoride in water a contaminant, as does the EPA, except when added purposely as an “additive” (NSF [47]). But additives are chemicals that treat water for sanitation, not to treat humans through internal ingestion. Chemical supplier data sheets also place liability on the end user.

 Standard 60 requires that all water additives be allowed to only 10% of any EPA maximum contaminant level MCL which for the fluoride contaminant developed for natural water containing calcium is 4 ppm. Unnatural fluoride has been infused typically to 1.2 ppm, 30% of the MCL. This evasion of the NSF toxicology rule is allowed in part by considering it as a normal ingredient in water [8]. As evident in the present article, labeling the fluoride anion alone as a water ingredient without consideration of the associated positive ions and its environment is incorrect. Natural fluoride in water is always accompanied with calcium from the dissolved salts. The EPA MCL was based on observations from natural calcium fluoride in water. Additional sources of industrial fluoride have now become considerable, and many water sources are very low in calcium content. Thus, the EPA MCL does not apply for industrial added fluoride or for many regions such as the soft water Pacific Northwest. A separate MCL is necessary for fluoride from industrial sources that also considers varying levels of interacting endogenous calcium.

NSF Standard 60 for water additives is not applicable to chemicals added to alter human tissue. The purpose of fluoridating water supplies is to affect teeth from ingestion and internal consumption of fluoride. Prescriptions have long been required for those who desire that children ingest sodium fluoride (Luride tablets) for perceived dental effect [48]. The tablets are not FDA approved to be taken internally but nevertheless still remain allowed by prescription. Total fluoride exposure from all sources must be monitored by prescription because ingested sodium fluoride rodenticide acts acutely through calcium sequestration, and subacute doses taken long-term of course cause cumulative pathology. Most prescriptions state that the drug is not to be used in areas where water contains 0.6 ppm fluoride or higher [48]. Dosage instructions in the *Physician's Desk Reference *[48] have varied from 1994–2013 and in 1998 state—Warning: Do not use Luride 1 mg tablets for children under age 3 or use in patients under age 6 when fluoride water content exceeds 0.3 ppm. Luride tablets in any strength are contraindicated for all age groups when fluoride content of drinking water exceeds 0.6 ppm. The 2013 issue has similar warnings for vitamins with added sodium fluoride. These restrictions attempt to minimize abnormal enamel fluorosis and other adverse pathology but does not consider the endogenous water calcium level, other widespread fluoride sources that equal or exceed the prescribed dose even when drinking water is devoid of fluoride (Lee [49]), and that fluoride ingestion is not FDA approved. Cities fluoridate water to 0.7 ppm or higher but do not announce that use of fluoride tablets should be discontinued or that systemic fluoride is a recognized neurotoxin in animals and man that accumulates abnormally and permanently into bone [8, 9]. Thus officials within the CDC, NSF, and EPA appear to be unaware that these agencies taken together recommend, certify, allow and describe the consumption of industrial fluorides from treated drinking water supplies. This perturbs the prescription process and interferes with public understanding that industrial fluorides are toxics and are not FDA approved for ingestion. 

The SDWA states “No National primary drinking water regulation may require the addition of any substance for preventive health care purposes unrelated to contamination of drinking water” (Graham and Morin [50]). Chlorination is used for sanitation. But fluorides are added to treat humans. This provision was specifically intended to prohibit the use of the SDWA as a means to impose artificial fluoridation of public water supplies throughout the U. S. (Graham and Morin [50], note 88, p. 213). Yet Federal and state agencies and chemical suppliers assign all liability to cities, which are typically the least informed of the risks and do not necessarily carry insurance that would cover fluoride-related litigation. A California law attempts to mandate “fluoride" (see Endnote 1) in public water supplies without listing the specific substance required. Since the toxicity of all compounds containing fluoride is determined by its ionic co-ingredient, the law is neither actionable nor a mandate. Funds for silicofluoridation are typically derived from tobacco tax money, dental insurance premiums, or directly from ratepayers. San Diego citizens voted twice against adding fluoride compounds into water but fluorosilicic acid infusions began there in 2011. 

Silicic acid has no EPA MCL so a standard of 16 ppm was created and deemed safe as an allowed maximum without conducting or soliciting toxicity studies that NSF documentation requires [47]. This is thus circular reasoning. NSF states that silicic acid infused to 0.8 ppm in fluoridated water is below a safe standard. But NSF helped determine the standard deemed to be safe without toxicity data to back it up. The EPA has authority to prohibit the intentional addition of any contaminant under the authority of the SDWA. Instead, a current EPA fact sheet on hazardous waste fluorosilicic acid describes its use as an ingestible dental prophylactic for use in water supplies (EPA [38]).

 The FDA requires a new drug application for any material containing HF intended for human ingestion [51]. Fluorosilicic acid typically contains 1.5–2% HF. The EPA fact sheet focused on the upper portion of the titration curve in Figure 2(b) where HF is negligible above pH 4.2 to imply the material is safe for long-term oral ingestion. However, as shown in this paper, fluoride converts to HF below pH 4 as in the acidic stomach and is contraindicated as a proposed human treatment. 

 The FDA ruled in 1975 that fluoride is not considered safe to add to foods (Sutton [40]). But fluoride drinking water regulations are still being revised by the U.S. Health and Human Services (HHS). The HHS and EPA joint recommendation in 2011 to lower the allowed fluoride level in water was a temporary response to requests made by the NRC. It is not expected that the FDA would approve soluble fluorides for ingestion because controlled human clinical trials data are required for review. It would be unethical to conduct human trials when industrial fluoride at blood levels typically found in residents of fluoridated cities is recognized as a neurotoxin, a non-physiologic mitogen ([9] page 337 Table  10-5), a general enzyme inhibitor, and a permanent bone perturbant during chronic consumption [8, 9]. This author submitted a petition to the FDA requesting a general ban on the sale or use of all industrial fluorides intended for internal ingestion by dissemination through public water supplies in the U.S. The petition was accepted for review in 2007 and remains pending (FDA [37]). 

## 4. Conclusions

This study indicates that industrial fluoride added to drinking water forms intact corrosive hydrofluoric acid under acidic conditions that prevail in the stomach of man (pH 1.5–3) and animals. Ingested fluoride from water enters the bloodstream as an artificial component, not a normal constituent, and disrupts intermolecular hydrogen bonding, forming interatomic hydrogen bonding. Fluoride influences calcium homeostasis. Accidental higher levels of fluoride known to cause acute lethality compare with calculated levels that would begin calcium precipitation at physiologic calcium concentrations in blood. The difference between the single oral acute fluoride dose of 60 mg/kg body weight and the lethal blood concentration of 2-3 ppm, calculated here and observed clinically in blood after accidental fluoride poisonings, may be due in part to fluoride elimination by kidneys and accumulation by bone during assimilation of the ingested oral dose. It is not possible to reach an acute lethal blood level of industrial fluoride from treated water unless there were an accidental overfeed. 1 ppm water leads typically to ~0.2 ppm blood fluoride. But only ~1 ppm blood levels cause a chronic form of congestive heart failure (found after hemodialysis with fluoridated water) and 2-3 ppm causes acute heart failure. 

 The infusion of industrial fluorosilicic acid with caustic sodium hydroxide into water supplies introduces sodium, that is not a component of fresh drinking water, plus fluoride without calcium. Sodium and fluoride are the ingredients used in rodenticides and in the prescription drug Luride which is not approved by the FDA for ingestion. Ingested sodium fluoride from treated water does not reduce caries either systemically at 0.2 ppm or topically from saliva at 0.02 ppm. Instead it increases the incidence of unsightly abnormal dental fluorosis hypoplasia in all treated cities. The policy adopted by the U.S. Public Health Service in 1950 remains encouraged by the trade organization the American Dental Association, dental insurance providers, and dental officials in the Oral Health Division. But none of these groups has authority to chemically treat public water supplies. The rationale for the infusions remains based on early observations that were not supported by careful experimentation using the scientific method. When examined in detail this proved to be an anecdotal incorrect correlation.

Federal law prohibits any requirement for substances added into water other than to sanitize water, regardless of ascribed benefit. And yet, plain water without added industrial fluoride is now scarce in U.S. public supplies. The decision to infuse industrial fluoride compounds into public water supplies to permeate the blood and organs of consumers with fluoride as an ingested dental prophylactic was an error that resulted in serious consequences including loss of life. Although many believe that the infusions decrease caries without causing systemic damage, the data reported here along with other published studies do not support the policy [1, 7–12, 26, 27, 30–35, 40, 41, 52–54]. Insidious effects that can occur on musculoskeletal, neurologic, reproductive, and endocrine systems from long-term ingestion of fluoride in water [8, 9, 11] and the cardiovascular effects discussed here emphasize the seriousness of fluoridation especially in soft water regions lacking antidote calcium. Also fluoride exposure is now from diverse sources. 

Adding substances in water that are unnatural, harmful, illegal, and ineffective in its stated purpose violates universally accepted consumers' and patients' rights of refusal. This is because fluoride at subsaturation levels is not easily filtered. A legal review described the policy as un-Constitutional (Balog [46]). Enamel hypoplasia and caries are not caused by absence of fluoride. Essentially all European countries do not fluoridate public water supplies but some do offer optional fluoridated salt that is not as extensively consumed as water. Opposition has been widely publicized in the U.S. (Abby Martin and RT News [55] and the documentary film *Fluoridegate* [56]), Canada, and the U.K. [52, 53, 57]. Citizens mostly vote against fluoridation but the SDWA should have been sufficient law to avoid the need for voting. The city of Portland, Oregon recently voted against fluoridation for the fourth time and remains untreated thus far. 61 cities in Nebraska voted against fluoridation over the period 2008-2009, effectively overruling a state mandate. The policy does not accommodate kidney dialysis patients and those who are normally fluoride-sensitive [12]. For all who object, the policy evades human decency.

## 5. Endnotes

1. Definition of terms fluoride, a fluoride, fluorides, fluoride mineral(s), mineral fluoride(s), fluorine, fluoride compound(s): In chemical nomenclature “ide” refers not only to the nonmetal anions but also to all binary compounds. *Fluoride* is the ion F^−^. It can be found naturally in minerals or dissolved from minerals as the free ion in water accompanied with cations with which it coexists. “A fluoride” refers to compounds containing fluoride including natural minerals and industrial compounds or to a single fluoride ion. “Fluorides” refer to any compounds containing fluoride, natural or otherwise. Fluoride minerals or mineral fluorides are the natural compounds that contain the fluoride ion.

*Fluorine *specifically refers only to pure elemental diatomic molecules of the atoms, F_2_. Even though commonly done in respected reference sources, it is incorrect to state that fluorine has a natural abundance on the earth's crust. It does not. Elemental fluorine does not exist in nature. It is also incorrect to state that fluoride in the earth's crust is a mineral. Natural sources that contain the fluoride ion are fluorite, cryolite, and fluorapatite [4]. Fluorite is found in metallurgical, ceramic, and acid grade with CaF_2 _content ranging from 60–97%. These are natural fluoride minerals or mineral fluorides. But there is no mineral called fluoride (even though “a fluoride” can refer to a natural fluoride mineral).

 The difference between industrial fluoride and fluoride minerals is chemically and biologically vast. Natural mineral fluorides are not absorbed well when ingested because of the natural metal cations that accompany fluoride. Having no biologic similarity at all with natural fluoride minerals, industrial manufactured fluoride compounds have cations that replaced those in the natural mineral. Sodium fluoride is an industrial, fully soluble, metal-nonmetal fluoride. Fluorosilicic acid is an industrial, fully soluble, metalloid-nonmetal fluoride. 

 The CDC website on fluoridation writes extensively that there is “no difference” between natural and industrial fluoride since the ion is itself identical in all materials. But the reactivity and toxicity of fluoride ion is determined by the positive cation company it keeps. Free fluoride ion in some water supplies as a contaminant is naturally present from natural fluoride minerals that exhibit low solubility. The equilibrium double-arrow natural partial dissolution of the insoluble solid into some waters is given by: CaF_2_(s)↔Ca^2+^(aq) + 2F^−^(aq). Industrial fluorides stripped of natural mineral cations lack antidote calcium and are fully assimilated from artificially treated water with insufficient calcium. The unnatural industrial fully soluble material reacts to completion, approximated as: H_2_SiF_6_ + 6NaOH → 6F^−^(aq) + 6Na^+^(aq) + H_4_SiO_4_(aq) + 2H_2_O. 

 Binary covalent compounds can be labeled fluorides when the fluorine atom is synthetically attached to a nonmetal atom as in fluorocarbons. These compounds, binary or not, do not contain either the fluoride charged ion F^−^ or the neutral fluorine F atom and are often labeled fluorinated substances rather than fluorides because of partial charge separation. The unnatural C-F bond is lipophilic.

2. The average lifespan for many animal species is reduced significantly by providing 1 ppm fluoridated water for their entire lives (Yiamouyiannis [11], Spittle [12]). Alligators died prematurely living in the treated water. Horses turn over whole body water quickly and perished from complications due to continuous exposure to fluorosilicic acid treated soft water over a 9 year period. Recent evidence suggests that aluminum treated silicofluoridated water in Southern California since 2007 appears to be a contributing factor in the increasing incidence of racehorse deaths in Southern California (upcoming editorial in *Fluoride*). 

No citizens have yet been exposed to industrial fluoride treated water for an entire human average lifetime of 75 years even though retained fluoride accumulates during chronic continuous exposure. Grand Rapids treated since 1945 could have individuals who have consumed fluoridated water for 68 years. It has long been known that lifetime (>60 years) consumption of water with 1 ppm natural fluoride in the absence of fluoride toothpaste causes fluoride to accumulate into bone to 4,000 mg/kg, a level associated with weakening of bone, making bone more subject to fracture (NRC, [9]). In 2000, there were 2.6 million medically treated non-fatal fall-related injuries (Stevens [58]) with medical care costs totaling 19 billion dollars. Consumption of 1 ppm industrial fluoride from drinking water together with fluoride toothpaste and other fluoride sources for 68 years would be expected to cause far larger fluoride incorporation. Besides possible shifts in the IQ of the population to a lower mean, bone fluoride accumulation could be associated with arthritis-type pain. The subset of the population with atherosclerosis would have more extensive fluoride incorporation into the aorta which is an inappropriate risk when cardiovascular disease is the nation's leading lethal disease entity. And 1% of the people would need to live with fluoride hypersensitivity by avoiding treated water supplies or by moving away from the area. 

Fluorides used as dental treatments in pastes and gels can be avoided. But fluoride added into water supplies is a burden. The ion compares in size to water molecules and is difficult to eliminate except with sophisticated procedures including high pressure reverse osmosis (~0.27 nm pore size), binding by specially prepared bone char, or distillation. Millions of dollars can be saved for any city by simply not purchasing waste fluorosilicic acid and instead treating caries differently with promotion of sufficient dietary calcium phosphate to help maintain strong teeth. Blood calcium homeostasis is a far more important consideration than are dental caries. Fluoridation equipment and fluoridation engineers can easily become involved in removing EPA contaminants from water instead of adding them into water. The presence of natural calcium fluoride in some waters should not be labeled “natural fluoridation”. Fluoridation refers to the intentional addition of fluoride compounds which are mostly soluble industrial synthetic fluoride toxic calcium chelators into water supplies to treat humans. Fluoridation needs to be revoked as a general public policy because the actual purpose of drinking water is to hydrate living cellular tissue with H_2_O. Daily amounts depend on level of physical activity, health conditions (diabetics require more water), personal taste, environmental and other factors.

3. The prevalence of Alzheimer's disease in the U.S. is 4-5 million people. The cost of care for Alzheimer's, chiefly for nursing care facilities, totals 2 billion dollars annually in the U.S. (*New England Journal of Medicine*, 2013 in press). 75% of Alzheimer's victims require nursing home care, compared to 5% without Alzheimer's. It is the 6th leading cause of death and has no cure or effective treatment. It is the most expensive in costs of care of any disease entity in the U.S. The EPA recently classified fluoride as a neurotoxin but this requires clarification. Fluoride minerals are not neurotoxins, because fluoride is not absorbed from ingested minerals. Free fluoride ion in drinking water can be so classified, but industrial fluoride sources are assimilated more readily than fluoride from hard water or from natural calcium fluoride. Blood-borne fluoride regardless of source is neurotoxic and should be avoided from any source by individuals with neurologic conditions such as autism and Alzheimer's disease. Brain pathology can produce symptoms that are not necessarily readily quantified but recent studies confirm that fluoride levels in blood directly correlate with measurable reduction in IQ (Xiang et al. [54]).
