# Corrigendum to “Physiologic Conditions Affect Toxicity of Ingested Industrial Fluoride”

**DOI:** 10.1155/2017/4239182

**Published:** 2017-05-30

**Authors:** Richard Sauerheber

**Affiliations:** ^1^Department of Chemistry, University of California, San Diego, La Jolla, CA 92037, USA; ^2^STAR Tutoring Center, Palomar Community College, San Marcos, CA 92069, USA

In the article titled “Physiologic Conditions Affect Toxicity of Ingested Industrial Fluoride” [1], there was an error in the fourth paragraph of section “3.3. Natural and Industrial Fluoride in Water.” The text reading “Fluoride accumulates from consumption in a 1 ppm fluoride water region, in the absence of other known sources, to 2,500 mg/kg in two years and to 3-4,000 mg/kg lifetime [9]” should be corrected to “Fluoride accumulates from consumption in a 1 ppm fluoride water region, in the absence of other known sources, to 2,500 mg/kg in twenty years and to 3-4,000 mg/kg lifetime [9].”
